# Factors Associated with Survival From Xp11.2 Translocation Renal Cell Carcinoma Diagnosis—A Systematic Review and Pooled Analysis

**DOI:** 10.3389/pore.2021.610360

**Published:** 2021-03-30

**Authors:** Yuqing Wu, Saisai Chen, Minhao Zhang, Kuangzheng Liu, Jibo Jing, Kehao Pan, Lihua Zhang, Bin Xu, Xiaoming Lu, Ming Chen

**Affiliations:** ^1^ Surgical Research Center, Institute of Urology, School of Medicine, Southeast University, Nanjing, China; ^2^ Department of Urology, Affiliated Zhongda Hospital of Southeast University, Nanjing, China; ^3^ Department of Pathology, Affiliated Zhongda Hospital of Southeast University, Nanjing, China; ^4^ Department of Urology, Yancheng Third People's Hospital, Yancheng, China; ^5^ Lishui People’s Hospital, Nanjing, China

**Keywords:** TFE3, kidney, Xp112 translocation renal cell carcinoma, survival, prognosis

## Abstract

**Purpose:** Xp11.2 translocation renal cell carcinoma (Xp11.2 tRCC) is a rare subtype of renal cell carcinoma (RCC), characterized by translocations of Xp11.2 breakpoints, involving of the transcription factor three gene (TFE3). The aim of our study was to comprehensively characterize the clinical characteristics and outcomes, and to identify risk factors associated with OS and PFS in Xp11.2 tRCC patients.

**Methods:** Literature search on Xp11.2 tRCC was performed using databases such as pubmed EMBASE and Web of Science. Studies were eligible if outcomes data (OS and/or PFS) were reported for patients with a histopathologically confirmed Xp11.2 tRCC. PFS and OS were evaluated using the univariable and multivariable Cox regression model.

**Results:** There were 80 eligible publications, contributing 415 patients. In multivariable analyses, the T stage at presentation was significantly associated with PFS (HR: 3.87; 95% CI: 1.70 to 8.84; *p* = 0.001). The median time of PFS was 72 months. In the multivariable analyses, age at diagnosis (HR: 2.16; 95% CI: 1.03 to 4.50; *p* = 0.041), T stage at presentation (HR: 4.44; 95% CI: 2.16 to 9.09; *p* < 0.001) and metastasis status at presentation (HR: 2.67; 95% CI: 1.12 to 6.41; *p* = 0.027) were all associated with OS, with a median follow-up time of 198 months.

**Conclusion:** T stage at presentation is the only factor that is associated with both PFS and OS in patients with Xp11.2 tRCC. Also, patients over 45 or with metastases are more likely to have poorer OS.

## Introduction

Since being listed as a new type of renal cell carcinoma (RCC) by the World Health Organization (WHO) in 2004 [1], Xp11.2 translocation renal cell carcinoma (Xp11.2 tRCC) has received wide attention around the world [2]. It’s a rare subtype characterized by several different chromosomal translocations of Xp11.2 breakpoints and involves the formatting of the transcription factor three gene (TFE3), leading to a fusion gene with a significant overexpression of TFE3 protein in tumor cells [3]. It’s reported that, compared with Xp11.2 tRCC, RCC associated with t (6; 11) (p21; q12)/TFEB gene fusions has similar epidemiology pathology, and genetics characteristics [4]. In 2016, WHO newly designated Xp11.2 tRCC as microphthalmia-associated transcription (MiT) family translocation RCC since both TFE3 and TFEB belong to MiT factor family [5].

Xp11.2 tRCC, which predominantly occurs in children and young adults, is more aggressive than other conventional RCC due to its advanced stages and invasive clinical courses in adults, regardless of its low incidence [6]. Microscopically, it is difficult for pathologists to distinguish Xp11.2 tRCC from other types of RCC. Although immunohistochemistry (IHC) can detect the overexpression of TFE3 protein that is involved in TFE3 gene, serving as the basic method for diagnosis of Xp11.2 tRCC, high false-positive rates and low predictive values were reported [7]. Thus, the fluorescent *in situ* hybridization (FISH) provides a way which is more accurate in identifying the TFE3 gene rearrangement with higher sensitivity [7].

However, the prognosis of Xp11.2 RCC is still controversial due to the low appearance of series with enough number of patients and the short follow-up period. Also, the rarity of this disease and its under-recognition lead to few articles on Xp11.2 tRCC progressing that have been published, most of which were performed in the form of single case report and small series, limiting the generation of definitive conclusions regarding clinical characteristics, risk factors, prognosis and treatment. Therefore, it is necessary to identify the OS and PFS, as well as the factors that are associated with prognosis in patients with Xp11.2 tRCC.

In this study, we have systematically reviewed the studies on Xp11.2 tRCC to comprehensively characterize the clinical characteristics and outcomes, and to identify risk factors associated with OS and PFS in Xp11.2 tRCC patients.

## Patients and Methods

### Search Strategy

The following databases were searched by October 2020: PubMed, EMBASE, Web of Science and other specialty meeting abstracts. The search terms used are as follow: (TFE3 OR XP11.2 OR MITF translocation) AND (renal cell carcinoma OR RCC). There were no limitations on the language or published time of studies. Reference lists were also checked for relevant articles. The most recent publications were chosen when they included updates to prior ones.

### Inclusion and Exclusion Criteria

Two reviewers (Wu and Liu) independently screened the identified abstracts for eligibility and full articles for detailed evaluation. Studies were eligible if outcomes data (OS and/or PFS) were reported for patients with a histopathologically confirmed Xp11.2 tRCC, which was diagnosed by TFE3-IHC or FISH. When the result of TFE3-IHC was different from FISH, the result of FISH was taken to decide the final diagnosis.

Studies were excluded if they were 1) lack of available outcomes data; 2) with ambiguous inclusion criteria; 3) reviews; 4) not performed in human. When same group of patients were reported from one institution in different studies, the most recent data were chosen.

### Data Extraction

Two authors (Wu and Zhang) independently extracted the following data from included articles if available: 1) first author; 2) year of publication; 3) age at Xp11.2 tRCC diagnosis; 4) patient gender; 5) laterality of tumor; 6) clinical presentations at diagnosis; 7) disease history; 8) pathologic grade; 9) stage of disease at diagnosis; 10) primary treatment; 11) adjuvant treatment; 12) prognostic outcomes. Disagreements were resolved through discussion. Disease history was defined as kidney disease that patients had before.

### Statistical Analysis

OS was defined as the time from the date of diagnosis of Xp11.2 tRCC to death. In the absence of confirmation of death, OS was censored at the last date when the patient was known to be alive. PFS was defined as the time interval between the date of surgery and date of disease recurrence or metastasis. Data on patient demographics, the characteristics of tumors, and treatment approaches were summarized using descriptive statistics. The associations between these factors and OS and PFS of Xp11.2 tRCC were evaluated using univariable and multivariable Cox regression models, and hazard ratios (HR) with 95% confidence intervals (95% CI) and *p* values are presented. Survival analyses of OS and PFS were generated using the Kaplan-Meier method, and the log-rank test was performed for the significance comparison. Generally, *p* < 0.05 (two-sided) was considered statistically significant. Statistical analyses on survival were performed using SPSS version 19.0 (SPSS, Chicago, IL).

## Results

### Search Results and Characteristics of the Included Studies

**FIGURE 1:**
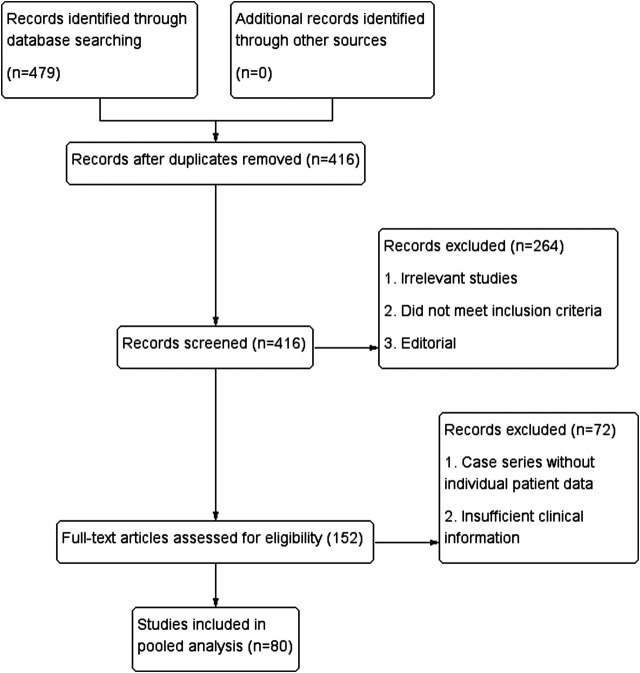
Flow diagram showing the selection process for the systematic review.

Our search strategy yielded a total of 479 articles. After the screen of abstracts, 152 articles were considered to be relevant reports. After reviewing these articles, we found 80 that reported histopathologically confirmed Xp11.2 tRCC and fulfilled the inclusion criteria (Figure 1), giving a total of 415 patients [2], [8–10], [11–20], [21–30], [31–40], [41–50], [51–60], [61–70], [71–86]. Baseline characteristics are summarized in Table 1.

**TABLE 1:** Patient and Tumor Characteristics at Xp11.2 tRCC Diagnosis.

Characteristics		No. of patients		%
Age, years				
Median			33	
Range			1–86	
Sex				
Male		176			43.67
Female		227			56.33
Location				
Right		167			57.99
Left		121			42.01
Clinical manifestations				
Symptomatic		129			57.33
Asymptomatic		96			42.67
Disease history				
Yes		21			30.00
No		49			70.00
Stage				
1		123			38.80
2		36			11.36
3		77			24.29
4		81			25.55
pT				
1		192			53.04
2		57			15.75
3		99			27.35
4		14			3.86
pN				
0		185			64.91
1		63			22.11
2		37			12.98
pM				
0		281			82.16
1		61			17.84
Fuhrman				
1		1			0.77
2		39			30.00
3		67			51.54
4		23			17.69
Primary treatment				
Surgery		406			98.78
No surgery		5			1.22
Type of surgery				
Radical nephrectomy		276			77.97
Partial nephrectomy		78			22.03
Adjuvant treatment				
None		131			48.52
Targeted therapy		74			27.41
Immune therapy		56			20.74
Chemotherapy		9			3.33

### Survival Analyses of OS and PFS

**FIGURE 2:**
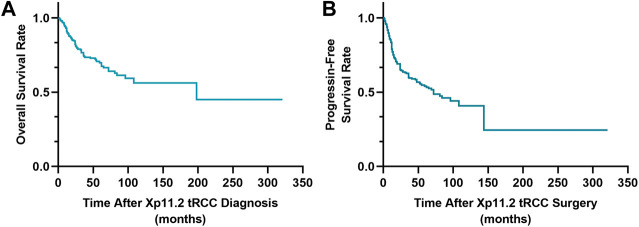
Survival curves of overall survival **(A)** and progression-free survival **(B)**.

The median time of PFS was 72 months (range: 1–321 months; Figures 2 and 198 months for OS (range: 1–321 months; Figure 2). The OS was 74.5 and 69.8% for 3-years and 5-years, respectively.

### Univariate and Multivariate Analyses for PFS and OS

In multivariable analyses, the T stage at presentation was significantly associated with PFS (HR: 3.87; 95% CI: 1.70 to 8.84; *p* = 0.001; Table 2), which was the only predictive factor for PFS. However, in the multivariable analyses for OS, age at diagnosis (HR: 2.16; 95% CI: 1.03 to 4.50; *p* = 0.041), T stage at presentation (HR: 4.44; 95% CI: 2.16 to 9.09; *p* < 0.001) and metastasis status at presentation (HR: 2.67; 95% CI: 1.12 to 6.41; *p* = 0.027) were all associated with OS (Table 3).

**TABLE 2:** Univariate and multivariate analyses for variables considered for progression-free survival (Cox proportional hazard regression model).

Variables		Univariate analysis		Multivariate analysis	
		HR (95% CI)	*p*	HR (95% CI)	*p*
Age (>45)		1.57 (1.08–2.29)	0.019	1.57 (0.63–3.96)	0.336
Gender (male)		1.48 (1.07–2.04)	0.019	1.40 (0.65–3.02)	0.391
Laterality (right)		0.68 (0.47–0.98)	0.048	0.66 (0.26–1.67)	0.380
Disease history (Yes)		1.37 (0.63–2.99)	0.426		
Symptomatic (Yes)		1.99 (1.29–3.05)	0.002	0.88 (0.30–2.57)	0.817
T stage at presentation (T3-T4)		5.26 (3.17–7.45)	<0.001	3.87 (1.70–8.84)	0.001
Metastasis (Yes)		6.01 (4.11–8.77)	<0.001	2.24 (0.64–7.81)	0.205
Fuhrman grade (G3-G4)		2.09 (1.28–3.47)	0.004	1.27 (0.43–3.75)	0.666
Surgery approach (RN)		4.87 (2.67–10.45)	<0.001	1.35 (0.37–4.99)	0.653
Adjuvant therapy	TT v none	6.45 (3.95–10.52)	<0.001		
	IT v none	1.55 (0.77–3.13)	1.224		
	CT v none	3.03 (0.90–10.14)	0.072		

TT, targeted therapy; IT, immune therapy; CT, chemotherapy.

**TABLE 3:** Univariate and multivariate analyses for variables considered for overall survival (Cox proportional hazard regression model).

Variables		Univariate analysis		Multivariate analysis	
		HR (95% CI)	*p*	HR (95% CI)	*p*
Age (>45)		1.90 (1.19–3.04)	0.007	2.16 (1.03–4.50)	0.041
Gender (male)		1.55 (1.03–2.33)	0.034	1.24 (0.64–2.38)	0.527
Laterality (right)		0.63 (0.39–1.01)	0.053		
Disease history (Yes)		0.70 (0.24–2.02)	0.508		
Symptomatic (Yes)		1.96 (1.16–3.43)	0.012	1.16 (0.59–2.25)	0.664
T stage at presentation (T3-T4)		5.79 (3.68–9.12)	<0.001	4.44 (2.16–9.09)	<0.001
Metastasis (Yes)		5.27 (3.29–8.45)	<0.001	2.67 (1.12–6.41)	0.027
Fuhrman grade (G3-G4)		1.11 (0.61–2.02)	0.741		
Surgery approach (RN)		3.6 (1.46–8.96)	0.005	3.95 (0.93–16.78)	0.063
Adjuvant therapy	TT v none	3.31 (1.78–6.17)	<0.001		
	IT v none	1.86 (0.86–3.99)	1.113		
	CT v none	4.51 (1.51–13.52)	0.007		

TT, targeted therapy; IT, immune therapy; CT, chemotherapy.

### Cox Analyses for PFS and OS for Patients in Larger Series

For studies including more than 12 patients, a separate analysis was conducted, involving 11 studies with 218 patients [24, 25, 37, 52, 55, 59, 61, 67, 72, 74, 80]. In multivariable analyses, the T stage at presentation was significantly associated with PFS (HR: 7.73; 95% CI: 3.09 to 19.33; *p* < 0.001; Supplementary Appendix Table 1). In the [23, 24, 37, 52, 59, 70], multivariable analyses for OS, T stage at presentation (HR: 7.30; 95% CI: 3.55 to 15.02; *p* < 0.001) and metastasis status at presentation (HR: 2.16; 95% CI: 1.10 to 4.26; *p* = 0.026) were associated with OS (Supplementary Appendix Table 2).

## Discussion

Xp11.2 tRCC is characterized by several chromosomal translocations involving the TFE3 gene on chromosome Xp11.2. TFE3 gene can be fused by several genes, such as ASPL and SFPQ [87]. However, the same ASPL-TFE3 fusion gene is involved in alveolar soft part sarcoma, which may lead to differences in clinical and morphological features and an imbalance of the translocation mechanism [88, 89]. Also, the function of chimeric TFE3 fusion proteins varies a lot, which may lead to the different histological features in Xp11.2 tRCC [90].

Studies focusing specifically on patients with Xp11.2 tRCC are rare and limited by small samples. This study is the first one to collect the previous relevant studies and investigate the prognostic factors for PFS and OS in patients with Xp11.2 tRCC to our knowledge.

After being recognized as a distinct entity by WHO in 2004, the diagnosis of Xp11.2 tRCC usually depend on microscopic and THE3-IHC. Although Wang et al. in 2017 suggested only part of patients with positive reaction to TFE3-IHC were eventually pathologically diagnosed with Xp11.2 tRCC by FISH assay [80]. Compared with cytogenetics, THE3-IHC is equipped with higher speed and sensitivity of diagnosis, the sensitivity of specificity of which were found to be 97.5 and 99.6%, respectively [89]. Thus, TFE3-IHC can be conducted for screening, and FISH can be conducted for verification.

In reported cases, there was an observed prevalence of females (56.33%), patients under 45 years old (77.67%) and right side (57.99%) from all 80 studies with 415 cases, which is in line with the previous studies with case series [14, 23, 24, 37, 52, 59, 70, 80]. Liu et al. reported 34 patients with Xp11.2 tRCC, where females accounted for 61.8% and people under 18 for 88.2%. Also, in a report by Qu et al. [70], female and right-side cases accounted for 18 and 17 of 30 cases, respectively. Same pattern can be found in rest studies [14, 23, 24, 37, 52, 80]. Since the occurrence of Xp11.2 tRCC involves the translocation of Xp11.2 chromosome, and compared with males, females have one more X chromosome, which may cause higher incidence of this disease. However, it’s still not clear why predominance existed in right side.

According to previous studies, Xp11.2 tRCC is more common in children and young adults under 45 years old (low incidence of 0.2–5.0%) [2, 37, 49], but it was found to be more aggressive in adults [91]. In the present study, we found that the age is an independent predictor for the OS in patients with Xp11.2 tRCC (HR: 2.16; 95% CI: 1.03 to 4.50; *p* = 0.041). However, no standard treatment has been raised and radical nephrectomy (RN) is the first choice if possible [92], which accounts for 77.4% of 305 cases with reported surgery approach in the present study. Meanwhile, RN was reported to have beneficial outcomes in the treatment of patients as well. Dai reported eight patients with the treatment of RN who had a median PFS of 32 months in 2018 [25]. In multivariate analyses in present study, there is no significantly association between surgery approach and prognosis in Xp11.2 tRCC patients, and there’s still lack of long-time follow-up due to its late recurrence [93]. In a study of Liu et al. in 2017, all nine patients were treated with partial nephrectomy (PN), with a median PFS of 51 months [57]. In previous studies, RN was reported to be associated with an increased risk of postoperative complications, and PN has been proposed to achieve a better overall survival result in patients with RCC [94, 95]. Thus, PN can be considered as a main approach in the treatment of Xp11.2 tRCC.

In this study, we found that pT status, which was reported to contribute to the advanced TNM stage [96], is significantly associated with both PFS and OS in Xp11.2 tRCC patients. Furthermore, it’s suggested that TNM stage is the most significant factors associated with poor prognosis in Xp11.2 tRCC patients, in the study with 34 patients of Liu in 2016 [59]. Meyer reported three patients who were in the late stage of Xp11.2 tRCC and had metastasis, ending up with an average time of 18 months for OS regardless of the adjuvant therapy [63].

With regard to the adjuvant treatment, there is still no data mentioning the most optimal or reliable treatment for each individual patient. In univariate analyses in the present study, patients treated with targeted therapy seemed to achieve a better result in both OS and PFS. Due to the close relationship between the choice of adjuvant treatment and TNM stage, in order to reach a more reliable result, we didn’t include the adjuvant treatment into the multivariable Cox regression models. Targeted therapy, immunotherapy and chemotherapy are the main options in included studies, which accounts for 53.2, 40.3 and 6.5% in the cases with reported adjuvant treatment in the present study. Targeted therapy, including sunitinib and sorafenib, was more likely to be chosen in patients with metastases [70]. Malouf reported two groups of patients with metastatic Xp11.2 tRCC, where one group with seven patients were treated with sunitinib, and the other one with 14 patients treated with immune therapy, showing a median PFS of 8 and 2 months respectively. Moreover, in the study of Wang in 2018, the DNA and RNA sequencing of Xp11 tRCC were reported, revealing novel gene fusions and presenting other potential targets for treatment [87]. Recent studies have shown that vascular endothelial growth factor receptor-targeted agents and mammalian target of rapamycin inhibitors play an important role effects in the treatment of metastatic TFE3 RCC [97–101]. Other targeted agents and immune checkpoint inhibitors are currently being tested and developed [102–104].

There are some limitations in our study. First, we included cases diagnosed with TFE3-IHC or FISH both in order to obtain a sufficient sample size, which may also lead to potential misdiagnoses. Second, because Xp11.2 tRCC is unpopular and underestimated in adults, and the information provided in the studies were insufficient and retrospective, the results should be interpreted discreetly. Third, due to the insufficient data in previous studies, when only the status of PFS was provided and evidence showed that the patient was alive, the survival data was converted to OS, which may not be precise. Fourth, age was considered as a prognosis factor for OS in analysis for all cases, but wasn’t a significant factor in the separate analysis for larger series, which can be attributed to the selection bias brought by case reports. Also, variance may exist due to the different settings from researchers in different cases. However, the results of the present study are still beneficial for the further research and understanding of the factors associated with survival from Xp11.2 tRCC.

In conclusion, T stage at presentation is the only factor that is associated with both PFS and OS in patients with Xp11.2 tRCC. Also, patients over 45 or with metastases are more likely to have poorer OS. Additional studies are still needed for the identification of potential targets for novel therapies.
