# Supplementary material for: Factors Associated with Survival From Xp11.2 Translocation Renal Cell Carcinoma Diagnosis—A Systematic Review and Pooled Analysis
Source: Pathol Oncol Res. 2021 Mar 30;27:610360. doi: 10.3389/pore.2021.610360 (PMC8262176; doi:10.3389/pore.2021.610360)
Supplement: Supplementary file 1 [file DataSheet1.PDF]

Appendix Table 1. Univariate and multivariate analyses for variables considered for progression-free survival in larger series (Cox proportional hazard regression model)

Variables		Univariate analysis		Multivariate analysis	
		HR (95% CI)	P	HR (95% CI)	P
Age (> 45)		1.50(0.82 to 2.77)	0.191		
Gender (male)		1.15(0.70 to 1.88)	0.593		
Laterality (right)		0.79(0.44 to 1.42)	0.432		
Disease history (Yes)		0.33(0.04 to 2.60)	0.295		
Symptomatic (Yes)		2.52(1.24 to 5.11)	0.011	0.79(0.33 to 1.89)	0.599
T stage at presentation (T3-T4)		10.76(6.13 to 18.88)	<0.001	7.73(3.09 to 19.33)	<0.001
Metastasis (Yes)		9.63(5.61 to 16.54)	<0.001	3.25(0.99 to 10.62)	0.052
Fuhrman grade (G3-G4)		1.70(0.65 to 4.46)	0.283		
Surgery approach (RN)		4.79(1.50 to 15.27)	0.008	87265.95(0.00 to 4.35E)	0.955
Adjuvant therapy	TT v none	9.23(4.20 to 20.25)	<0.001		
	IT v none	2.03(0.76 to 5.42)	0.156		
	CT v none	1.94(0.24 to 15.56)	0.534		

Appendix Table 2. Univariate and multivariate analyses for variables considered for overall survival (Cox proportional hazard regression model)

Variables		Univariate analysis		Multivariate analysis	
		HR (95% CI)	P	HR (95% CI)	P
Age (> 45)		1.89(0.91 to 3.94)	0.088		
Gender (male)		0.97(0.52 to 1.80)	0.921		
Laterality (right)		0.73(0.38 to 1.41)	0.344		
Disease history (Yes)		0.29(0.04 to 2.10)	0.215		
Symptomatic (Yes)		1.76(0.81 to 3.80)	0.151		
T stage at presentation (T3-T4)		8.90(4.63 to 17.09)	<0.001	7.30(3.55 to 15.02)	<0.001
Metastasis (Yes)		5.37(2.79 to 10.31)	<0.001	2.16(1.10 to 4.26)	0.026
Fuhrman grade (G3-G4)		0.79(0.32 to 1.91)	0.596		
Surgery approach (RN)		2.69(0.83 to 8.66)	0.098		
Adjuvant therapy	TT v none	4.14(1.77 to 9.68)	0.001		
	IT v none	1.79(0.65 to 4.89)	0.257		
	CT v none	2.03(0.25 to 16.34)	0.504		
